# Ultrasonic-Assisted Nanoparticle Engineering to Enhance the Extraction Efficiency and Sensory Quality of Saudi Coffee

**DOI:** 10.3390/foods14162811

**Published:** 2025-08-13

**Authors:** Sameh A. Ahmed, Faisal S. Al-Amro, Yaser M. Alahmadi

**Affiliations:** 1 Department of Pharmacognosy and Pharmaceutical Chemistry, College of Pharmacy, Taibah University, AlMadinah AlMunawarah 30001, Saudi Arabia; 2 Department of Pharmacy Practice, College of Pharmacy, Taibah University, AlMadinah AlMunawarah 30001, Saudi Arabia; yahmadi@taibahu.edu.sa

**Keywords:** Saudi coffee, nanoparticles, ultrasonic-assisted processing, caffeine, total phenols

## Abstract

Background: Saudi coffee, made from Khawlani beans, is known for its sweeter, less acidic flavor and rich content of bioactive compounds. However, traditional preparation methods are time consuming and inefficient in extracting these compounds, limiting their global appeal. This study introduces an ultrasonic-assisted nanoparticle preparation technique to enhance the extraction efficiency, chemical profile, and sensory quality of Saudi coffee. The method aims to overcome limitations of traditional grinding by reducing the particle size while preserving key bioactive compounds. Methods: Finely ground coffee was subjected to ultrasonic processing at optimized parameters 450 W (60% of 750 W output), with 10 min of pulsed sonication to produce nanoparticles. These were characterized using SEM, FT-IR, XRPD, and particle size analysis. Comparative chemical analysis (caffeine, total phenols) and sensory evaluation were conducted against regular Saudi coffee. Results: Ultrasonication reduced the particle size to ~101 nm, significantly enhancing caffeine (from 0.54 to 3.21 mg/g) and phenolic content (from 426.7 to 1825.3 µg GAE/g). Solubility also increased from 40.7% to 75.9%. Sensory tests showed an improved aroma, mouthfeel, and flavor. These improvements are attributed to an enhanced extraction and surface area at the nanoscale. Conclusion: Ultrasonic-assisted nanoparticle technology significantly improves the physicochemical and sensory properties of Saudi coffee. This approach offers a fast, scalable, and eco-friendly method for quality enhancement, positioning Saudi coffee for greater global competitiveness.

## 1. Introduction

Saudi coffee, traditionally prepared from Khawlani coffee beans cultivated in the Jazan region of Saudi Arabia, is renowned for its distinctive flavor, characterized by a sweeter, less acidic profile compared to regular coffee varieties [1,2]. These Arabica beans are typically medium roasted and often infused with regional spices such as cardamom, saffron, and cloves, creating a culturally rich and complex beverage [2]. Despite its appealing sensory attributes and high content of bioactive compounds, the global expansion of Saudi coffee has been hindered by preparation challenges, particularly the inefficient extraction of flavor and health-promoting constituents. Conventional brewing requires prolonged boiling due to the physical characteristics of the coffee particles, leading to time-consuming and inconsistent extraction [3].

Nanotechnology offers innovative solutions for improving the functional and sensory properties of food and beverages by reducing material dimensions to the nanoscale. At this scale, the increased surface-area-to-volume ratio enhances the solubility, stability, and bioavailability of key compounds [4,5]. In coffee processing, particle size reduction through nanotechnology can significantly improve the release of caffeine, phenolic compound, and aromatic molecules, thus shortening the extraction time without compromising sensory quality [6].

Several nanotechnology-based techniques have been explored in coffee applications, including nano-grinding, nano-dispersion, nanofluid systems, and nano-crystallization. Nano-grinding improves extraction efficiency by increasing the surface area but often requires high-cost equipment such as ball mills and is limited by nanoparticle agglomeration due to high surface energy [7,8]. Nanofluids—colloidal systems in which nanoparticles are suspended in a base liquid—can enhance thermal conductivity and extraction kinetics but may alter traditional flavor profiles and are associated with high production costs and stability issues [9]. Similarly, nano-dispersion techniques enhance the bioavailability of active compounds but can lead to over-extraction or unbalanced sensory outcomes, potentially diminishing consumer acceptance [10]. Nano-crystallization methods further improve compound uniformity and solubility but face challenges related to cost, scalability, and nanoparticle instability during storage [11].

Among these approaches, ultrasonic-assisted nanoparticle preparation has gained traction in the food and beverage industry as an efficient and sustainable method. High-frequency ultrasound induces cavitation, which disrupts cell structures and enhances mass transfer, improving the extraction of bioactive compounds such as caffeine and antioxidants [12,13]. Compared to conventional methods, ultrasonication offers advantages such as a reduced processing time, minimal thermal degradation, and enhanced particle dispersion and stability [14]. Moreover, this method aligns with sustainability goals by reducing solvent and energy consumption [15]. The ability to tailor nanoparticle size and properties also opens new avenues for customizing flavor profiles and product performance.

Although ultrasonic-assisted techniques have shown promise in general coffee processing, their application in Saudi coffee has not been thoroughly investigated. Given the unique compositional and cultural value of Khawlani beans, this study aims to develop and optimize an ultrasonic-assisted nanoparticle preparation method specifically for Saudi coffee. The objectives are to improve extraction efficiency, enhance the content and solubility of bioactive compounds, and evaluate the impact on sensory attributes. This study also compares regular and nano-prepared Saudi coffee using comprehensive physicochemical and sensory analyses.

Ultimately, the proposed outcome of this research is to modernize the processing of Saudi coffee using advanced nanotechnology while preserving its traditional essence, thereby increasing its global appeal and economic value.

## 2. Materials and Methods

### 2.1. Sample Collection and Preparation

Coffee beans from the Khawlani variety were purchased the local market in Jazan, Saudi Arabia, then roasted with a medium roast technique, and sorted according to their quality. The beans were cleaned of impurities and dried for 4 h. The desiccated beans were crushed with a Moulinex^®^ commercial mill (Ecully, France) at two settings: rough and moderate at 22,000 rpm for 30 min with 2 min breaks alternating with 10 min pauses, to decrease the size of the particles. The average particle size of each grinding level was determined based on the granulometric distribution according to Colombian Technical Standard NTC 2441 [16]. A particle size range of 501 to 700 μm was employed for medium grinding, while 701–900 μm was used for coarse grinding.

### 2.2. Coffee Nanoparticle Preparation

The finely ground coffee particles were subjected to ultrasonic-assisted milling to transform them into nanoparticles. At first, 5 g of finely ground coffee beans was added to 20 milliliters of deionized water. Sono-emulsification was performed with a 24 kHz Vibra-Cell™ Ultrasonic Liquid Processor from Sonics & Materials, Inc., located in Newtown, CT, USA. The setup included a power generator, a sealed converter, and a horn microtip. The generator produces a frequency of 24 kHz, and the converter converted this electricity into mechanical vibrations. The internal transducer in the converter contained piezoelectric lead zirconate titanate (PZT) crystals that would expand and contract with the presence of alternating current. The 22 mm diameter horn tip conveyed the vibrations to the coarse emulsion it was submerged in. The ultrasonic waves had an adjustable amplitude, reaching a maximum power output of 750 W. Amplitude was indicated as a percentage of the maximum, with the horn microtip system restricted to a 70% operating amplitude because of the stronger cavitation effect generated by the more concentrated ultrasonic field, in contrast to the traditional horn tip. This study examined ultrasonic processing parameters, which consisted of amplitude level (%) and processing time (seconds). During sonication, a pulsed mode with 15 s of operation followed by 10 s of rest was used, resulting in a smaller particle size compared to continuous sonication. Various milling parameters were adjusted to obtain the most minimal particle size. A particle size analyzer was used to monitor the particle size constantly. The coffee nanoparticles were subsequently freeze dried to facilitate the quick production of instant coffee drinks.

### 2.3. Characterization of Coffee Nanoparticles

Different methods were employed to characterize coffee nanoparticles. Particle size and zeta potential were determined by a Microtrac S3500 analyzer (Microtrac Inc., Montgomeryville, PA, USA) after diluting the coffee nanoparticle suspension tenfold. The morphology and surface characteristics of the nanoparticles were examined using scanning electron microscopy (SEM, LEO 1530, Carl Zeiss SMT Inc., Oberkochen, Germany). SEM imaging was conducted at 12.50 kV with a 50 µm scale bar and an LFD detector. X-ray powder diffraction (XRPD) was employed for studying the crystalline structure of the nanoparticles. XRD patterns were generated using a Rigaku Miniflex II equipment, scanning from 10 to 60° at a rate of 0.04°/min. Three trials were performed for each test. Furthermore, FT-IR spectroscopy (FT-IR, Shimadzu IR Affinity, Kyoto, Japan) combined with ATR was used to detect changes in the molecular composition of the nanoparticles.

### 2.4. Determination of pH, Water Content, and Solubility

A digital pH meter was utilized to measure the pH of both the Saudi coffee nanoparticles and regular Saudi coffee extracts. To prepare a solution with a concentration of 1% *w*/*v*, 500 mg from each was dissolved in 50 milliliters of deionized water. The pH meter electrode was placed in the solution and left to stabilize for 60 s before the measurement was taken.

The solubility of Saudi coffee nanoparticles and regular Saudi coffee was measured in deionized water. A total of 500 mg of each extract was added in excess to 10 mL of deionized water and mixed for 12 h at a constant temperature (25 ± 0.5 °C) using an IKA^®^ KS 260 B shaker (Staufen, Germany) at a speed of 100 rpm. Next, the blends were spun in a centrifuge at a speed of 10,000 rpm for a duration of 10 min (Centrifuge Z 206 A; Hermle Labortechnik GmbH, Wehingen, Germany). The liquid above the sediment was passed through a 0.45 µm syringe filter, and a 0.1 mL sample was taken and mixed with deionized water. Solubility was determined based on the total dissolved solids (TDSs), calculated from the integrated area of UV-absorbing compounds detected at 254 nm, which primarily include caffeine, phenolic compound, and other water-soluble constituents of coffee. Solubility results were reported as the mean ± standard deviation (S.D.) of three replicates for each extract. Approximately 1 g of Saudi coffee nanoparticles and regular Saudi coffee were each weighed and put into a previously weighed moisture dish to determine their water content. Afterward, the samples were dried in a laboratory oven at a temperature of 105 °C for an entire day to remove all traces of water. After drying, the samples were transferred to a desiccator and allowed to cool for 30 min before being reweighed. The water content was calculated by comparing the weight of the sample before and after being dried. To find the water content percentage, the initial weight was multiplied by 100 and divided by the final weight; then, the initial weight was subtracted.

### 2.5. Regular Saudi Coffee Preparation

For comparison with the nanoparticle-prepared coffee, regular Saudi coffee was brewed using medium-ground Khawlani beans under typical domestic preparation conditions based on regional brewing standards. A total of 1 g of ground coffee was added to 100 mL of deionized water (1:100 *w*/*v* ratio). The mixture was heated in a traditional Arabic coffee pot over a medium flame until it reached a temperature of 95 ± 2 °C, then maintained just below boiling for 15 min. The brew was then allowed to settle for 3 min, and the upper clear portion was decanted for sensory and chemical analysis. All brewing was performed in triplicate under identical conditions.

### 2.6. Sensory Evaluation of Saudi Coffee Nanoparticles and Regular Coffee Extracts Procedures

The sensory evaluation of Saudi coffee nanoparticles and regular Saudi coffee extract was conducted by a trained panel of forty-three judges (twenty-two males and twenty-one females). The panel selection adhered to the guidelines outlined in ISO 8586-1 [17]. All assessors underwent pre-evaluation training to develop a unified sensory vocabulary specific to coffee beverages. Evaluation criteria included aroma, taste (encompassing bitterness), aftertaste, acidity, mouthfeel, and overall impact, and these were scored on a 0–5 scale according to ISO 11,035 [18]. A score of 3.0 was considered “good,” 3.5 “very good,” 4.0 “excellent,” and above 4.5 “outstanding.” Sensory tests were performed for both hot and cold coffee samples, each presented in triplicate at room temperature. The results were statistically analyzed using one-way ANOVA to determine the significance of differences between regular and nanoparticle coffee formulations.

### 2.7. Determination of Caffeine Content

The caffeine levels in Saudi coffee nanoparticles and regular Saudi coffee were measured in a previous study [19]. In this study, The caffeine levels in Saudi coffee nanoparticles and traditional Saudi coffee were measured using a Shimadzu Prominence series HPLC system with specific components. The system was managed using Shimadzu LC Solution software (version 1.21 SP1, Shimadzu, Japan). Prior to analysis, all samples and standards were filtered using 0.2 µm Millipore filters to remove particles. Isocratic chromatographic separation was carried out using a Thermo BDS Hypersil C18 analytical column with dimensions of 150 mm × 4.6 mm and a particle size of 5 μm. The separation process occurred at 25 °C in a room, with a mobile phase of methanol to water in a 30:70 (*v*/*v*) ratio. The flow rate of the mobile phase was set at 1.0 mL/min. Prior to usage, the mobile phase underwent thorough filtration and degassing through sonication. An Ultrasons-HD ultrasonic cleaner from Selecta, Barcelona, Spain, was utilized for degassing.

### 2.8. Determination of Total Phenolic Content

Total phenolic measurements in Saudi coffee nanoparticles and regular Saudi coffee were measured by the Folin–Ciocalteu method [20]. Every sample was mixed with deionized water in a 1:100 ratio. Next, 90 µL of deionized water, 15 µL of the diluted sample, 7.5 µL of Folin–Ciocalteu reagent (Sigma-Aldrich, Saint Louis, MO, USA), and 22.5 µL of 7.5% (*w*/*v*) sodium carbonate solution were added to each well of a microplate. Next, an additional 15 µL of deionized water was included. The samples were allowed to equilibrate at room temperature for 2 h before measuring the absorbance at 750 nm. Three measurements were taken for each sample, with the overall phenolic content being stated as grams of gallic acid equivalents per liter of solution.

## 3. Results

### 3.1. Optimization of Ultrasonic-Assisted Nanoparticle Preparation

Optimizing ultrasonic parameters like amplitude intensity, power output, and processing time is crucial in producing Saudi coffee nanoparticles. Regulating these factors is crucial for achieving the intended particle size, ensuring uniform distribution, and maintaining the structural stability of bioactive substances. Real-time dynamic light scattering (DLS) monitoring during sonication allowed prompt parameter adjustments, improving nanoparticle size control.

To optimize the ultrasonic-assisted nanoparticle preparation process, a series of systematic screening experiments were performed to evaluate the effects of amplitude intensity, power output, and processing time on the particle size, zeta potential, and dispersion uniformity of the Saudi coffee nanoparticles. The amplitude intensity varied from 20% to 90%, and its impact on particle size and the polydispersity index (PDI) was monitored using DLS. It was observed that increasing the amplitude from 20% up to 60% led to a progressive decrease in the average particle size and a notable reduction in the PDI, indicating improved uniformity and stability of the nanoparticle dispersion. Amplitude levels beyond 60% produced excessive cavitation energy, which resulted in localized overheating and partial aggregation, thereby compromising nanoparticle integrity. Thus, a 60% amplitude was identified as being optimal for producing uniformly sized particles of below 100 nm, with minimized aggregation and enhanced size control.

The power output of the ultrasonic processor was similarly evaluated in the range of 100 W to 750 W. Higher power levels strengthened cavitation, effectively disrupting aggregates and reducing the particle size. At 750 W, the most pronounced size reduction was achieved without evidence of compound degradation, as confirmed by zeta potential stability and color consistency. However, power levels above this threshold were avoided to minimize thermal stress. To prevent overheating during high-power sonication, the processor was equipped with an ice-cooled Vibra-Cell system, which helped to maintain the sample temperature within a safe range for bioactive compound preservation.

The processing time was optimized at between 2 and 20 min, using both continuous and pulsed sonication modes. A 10 min sonication period, applied in a pulsed mode (15 s ON/10 s OFF), was found to be ideal for achieving particle sizes below 100 nm and improving zeta potential stability. Durations under 6 min were ineffective, while those over 15 min caused re-agglomeration and reduced dispersion stability. The use of pulsed-mode sonication allowed for intermittent cooling, helping to preserve temperature-sensitive compounds and avoiding the degradation often associated with prolonged continuous ultrasonication.

Based on these experiments, the final optimized conditions selected for nanoparticle preparation were as follows: 450 W (60% of the maximum 750 W output), with 10 min of pulsed sonication. These parameters provided the best balance between particle size reduction, sample stability, and the preservation of coffee bioactive compounds.

### 3.2. Particle Size Analysis

**Figure 1:**
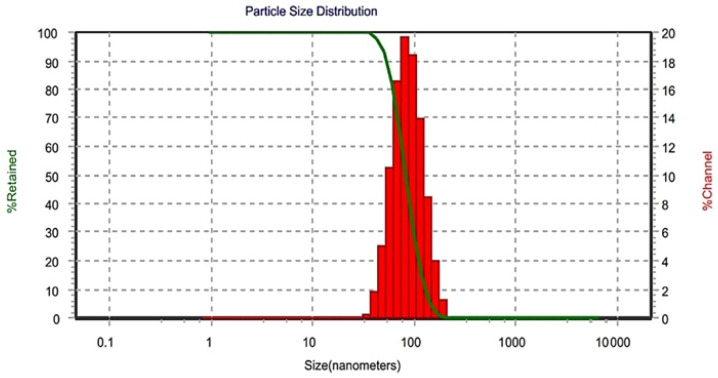
Particle size distribution of Saudi coffee nanoparticles produced via ultrasonic-assisted processing at 60% amplitude, 750 W, for 10 min in pulsed mode. The red bars represent the percentage of particles retained within each size channel (%Channel), while the green line represents the cumulative percentage of particles retained (%Retained). It is well well-known format for this type of figure as obtained from the instrument.

The size distribution of Saudi coffee nanoparticles made with the help of an ultrasonic-assisted method is depicted in Figure 1. According to the test findings, the Saudi coffee nanoparticles following the ultrasonic procedure displayed an average particle size of 101.0 nm, with a polydispersity index (PDI) of 0.254, indicating a relatively uniform size distribution. To further characterize particle size homogeneity, the D10, D50, and D90 values were calculated. The results showed that D10 = 73.4 nm, D50 = 98.7 nm, and D90 = 131.2 nm, indicating that 80% of the particles fall within a relatively narrow range of 73–131 nm. This distribution supports the observation of effective particle size control and narrow dispersion being achieved through ultrasonic-assisted nanoparticle processing.

In addition to size distribution, zeta potential measurements were conducted to assess colloidal stability. The Saudi coffee nanoparticles exhibited a zeta potential of −11.2 ± 1.2 mV, indicating moderate electrostatic repulsion and acceptable suspension stability. This negative surface charge reduces the likelihood of nanoparticle aggregation by promoting repulsive forces between particles. By contrast, the regular Saudi coffee extract displayed a significantly lower zeta potential of −2.6 ± 0.7 mV, suggesting weak colloidal stability and a higher propensity for sedimentation. These findings further support the effectiveness of ultrasonic-assisted processing in improving both particle size uniformity and dispersion behavior.

### 3.3. Particle Morphology

**Figure 2:**
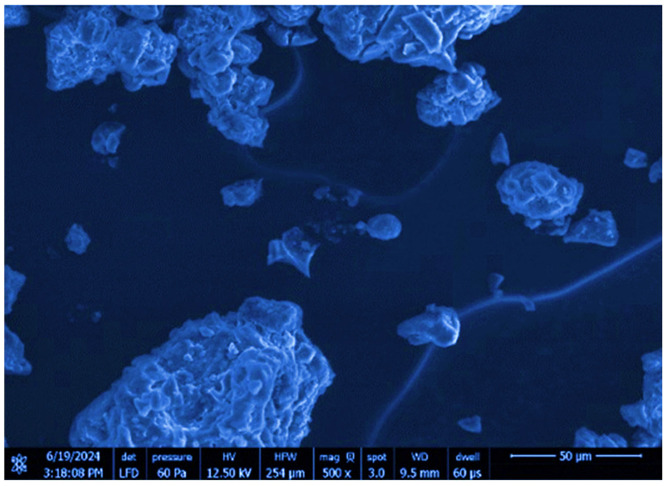
Scanning electron microscopy (SEM) image showing the surface morphology of Saudi coffee nanoparticles prepared using ultrasonic-assisted processing.

Scanning electron microscopy (SEM) was utilized to characterize the surface morphology of Saudi coffee nanoparticles. The analysis with a scanning electron microscope was performed using an LFD sensor at an accelerating voltage of 12.50 kV, as depicted in Figure 2. The picture was taken with a 500× zoom level and a 50 μm scale bar was included. According to the SEM findings, the particles show an uneven shape with varying particle sizes, a common characteristic of nanoparticles produced through methods such as ultrasonic-assisted synthesis. The uneven particle sizes seen in nanoparticle systems are likely caused by particles clustering together, which is a common occurrence due to their high surface energy. The nanoparticles in the SEM image appear as irregular shapes and agglomerates, indicating their tendency to cluster and form larger, uneven aggregates.

**Figure 3:**
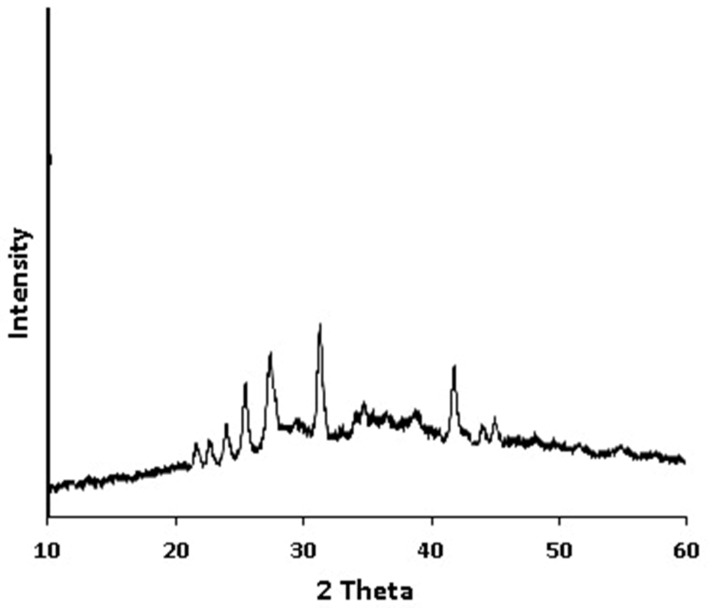
X-ray powder diffraction (XRPD) pattern of Saudi coffee nanoparticles prepared via ultrasonic-assisted processing. Distinct peaks around 25°, 27°, 32°, and 36° 2θ.

As is shown in Figure 3, the XRPD pattern of Saudi coffee nanoparticles exhibits distinct peaks that indicate the partial crystallinity of bioactive components. It revealed distinct diffraction peaks at approximately 25°, 27°, 32°, and 36° 2θ, suggesting the presence of partially crystalline structures. These peaks are consistent with the semi-crystalline nature of certain bioactive and structural components in coffee. Specifically, the peak around 27° may be attributed to caffeine, which is known to exhibit sharp crystalline reflections in this region. The peak near 25° could correspond to cellulose I or cellulose-derived structures, commonly retained in plant-based nanoparticle materials. Peaks at 32° and 36° may indicate the presence of chlorogenic acid or other polyphenolic residues, which have been reported to contribute weak but distinct crystalline signals in natural extracts. These findings suggest that while the nanoparticles retain some crystalline order, likely from inherent plant matrices and bioactive compounds, the overall structure remains predominantly amorphous, which may favor higher solubility and bioavailability.

### 3.4. Particle Molecular Structure Analysis

**Figure 4:**
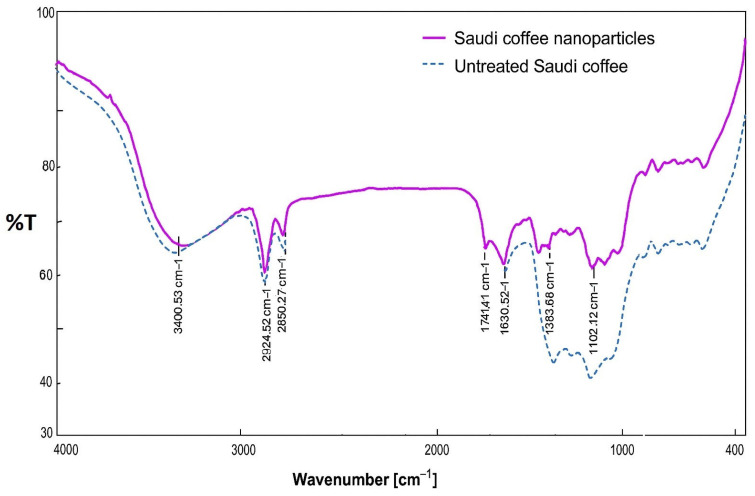
Fourier-transform infrared (FT-IR) spectra of Saudi coffee nanoparticles (solid line) and untreated Saudi coffee (dotted line).

FT-IR spectroscopy, which is frequently utilized to examine molecular vibrations and detect functional groups in substances, was used to investigate the molecular makeup of nanoparticles derived from Saudi coffee and untreated coffee. The FT-IR spectra of Saudi coffee nanoparticles and untreated coffee (Figure 4) reveal enhanced functional group visibility following ultrasonic processing, enabling comparative interpretation of molecular alterations induced by ultrasonic treatment. The broad absorption peak at around 3400 cm^−1^ corresponds to O–H stretching, indicative of hydroxyl groups commonly present in chlorogenic acids and polyphenols. This peak appears to be more intense with the nanoparticles, suggesting the enhanced exposure or release of these groups. The peaks observed at 2924 cm^−1^ and 2850 cm^−1^ are assigned to C–H stretching vibrations, typical of alkyl chains in caffeine and diterpenes. The peak at 1741 cm^−1^ corresponds to C=O stretching, likely derived from carboxylic acid groups in caffeic acid derivatives. Noticeable bands at 1630–1650 cm^−1^ and 1459 cm^−1^ represent the C=C stretching of aromatic rings, which can be associated with both caffeine and phenolic structures. The signal at 1378 cm^−1^ reflects C–N stretching, consistent with the presence of alkaloids such as caffeine. A prominent peak at 1102 cm^−1^ is assigned to C–O stretching, indicative of ether and ester linkages commonly found in chlorogenic acid esters. The comparison with untreated coffee shows no new peaks, suggesting that ultrasonic treatment did not degrade the core functional groups, but rather intensified the spectral features due to the improved accessibility and concentration of bioactive compounds in the nanoparticle form. These results support the preservation and potential enhancement of functional constituents, such as caffeine, chlorogenic acid, and other polyphenols, in the nanoparticle preparation, contributing to its improved functional and sensory qualities.

### 3.5. Determination of pH, Water Content, and Solubility

The pH data indicate that Saudi coffee nanoparticles (4.65 ± 0.07) exhibit significantly greater acidity than the regular Saudi coffee extract (5.76 ± 0.10) (*p* < 0.001), as shown in Table 1. The lower pH likely results from a reduced particle size, which increases the surface area and releases more acidic compounds like chlorogenic, quinic, and caffeic acids. Ultrasonic-assisted processing enhances the disruption of the coffee structure, promoting the release and availability of these acidic components. Previous studies have reported similar findings, where ultrasonication improved the extraction of organic acids from plant materials, resulting in a reduced pH due to greater exposure of dissociable acidic moieties [13]. This increased acidity may contribute positively to the flavor profile and enhance the physicochemical stability of the nanoparticle formulation.

Regarding moisture content, the regular Saudi coffee extract exhibited a significantly higher value (5.32 ± 0.15%) compared to the nanoparticle formulation (4.37 ± 0.12%) (*p* < 0.01). The reduced water content in nanoparticles could be attributed to structural modifications induced by sonication, which may reduce the capacity of particles to retain moisture. A lower moisture content may contribute to improved shelf stability and lower microbial susceptibility, favoring longer-term storage and formulation stability.

The solubility of Saudi coffee nanoparticles was markedly enhanced, reaching 75.88 ± 5.25% versus 40.70 ± 3.45% for the regular extract (*p* < 0.001). The increase is likely due to smaller particles and a greater surface area, improving water interaction and solute dispersion. Ultrasonic-assisted nanoparticle preparation facilitated the breakdown of larger aggregates, improving the availability and dissolution of active compounds. This significant enhancement in solubility may be especially advantageous in pharmaceutical, nutraceutical, and functional food applications, where bioactive compound solubility is a key determinant of efficacy and absorption.

### 3.6. Determination of Total Phenolic Content and Caffeine Content

Saudi coffee nanoparticles exhibited a significantly higher caffeine content of 3.21 ± 0.09 mg/g compared to 0.54 ± 0.05 mg/g in regular Saudi coffee (*p* < 0.001), as shown in Table 1. This enhancement is likely attributed to the ultrasonic-assisted extraction, which facilitates the improved release and solubilization of caffeine from the coffee matrix, yielding a more concentrated product. Similarly, the total phenolic content of the nanoparticles was significantly greater, measuring 1825.3 ± 72.8 μg GAE/g, in contrast to 426.7 ± 48.4 μg GAE/g for the regular extract (*p* < 0.001). This substantial increase suggests that ultrasonic cavitation, combined with particle size reduction, enhances the accessibility and extraction efficiency of phenolic compounds. The smaller nanoparticle size leads to greater surface area exposure, thereby increasing the yield of bioactive constituents. Collectively, the observed increases in caffeine and phenolic content highlight the efficacy of ultrasonic-assisted nanoparticle formation in enriching Saudi coffee with functional compounds of potential nutritional and pharmaceutical interest.

### 3.7. Sensory Evaluation of Saudi Coffee Nanoparticles and Regular Saudi Coffee Extracts

The sensory evaluation revealed that Saudi coffee nanoparticles scored significantly higher (*p* < 0.01) across all attributes compared to regular Saudi coffee extracts for both cold and hot beverage formats (Table 2). For aroma, nanoparticles were rated 3.9 ± 0.3 (cold) and 4.2 ± 0.2 (hot), indicating a very good to excellent aromatic quality, whereas regular coffee scored lower at 2.5 ± 0.4 and 3.1 ± 0.3, respectively. Regarding taste, the nanoparticle samples received 3.6 ± 0.4 (cold) and 4.0 ± 0.3 (hot), compared to 2.3 ± 0.5 and 2.8 ± 0.4 for the regular samples, highlighting a more robust flavor perception in the nanoform.

In terms of aftertaste, the nano-formulation again outperformed the regular coffee, scoring 3.4 ± 0.3 (cold) and 3.9 ± 0.3 (hot) versus 2.1 ± 0.4 and 2.7 ± 0.3, suggesting a more pleasant lingering sensation. Acidity, often linked to perceived brightness or freshness in coffee, was rated 3.2 ± 0.4 (cold) and 3.7 ± 0.2 (hot) for nanoparticles, which was significantly higher than 2.0 ± 0.3 and 2.5 ± 0.3 for the regular samples. For mouthfeel, nanoparticle coffee scored 3.8 ± 0.3 (cold) and 4.1 ± 0.2 (hot), suggesting a smoother, more velvety texture, while regular coffee scored 2.3 ± 0.4 and 2.9 ± 0.3.

Finally, for overall impact, the most integrative measure of sensory quality, Saudi coffee nanoparticles achieved 3.6 ± 0.3 (cold) and 4.3 ± 0.2 (hot), significantly exceeding the regular coffee ratings of 2.4 ± 0.4 and 2.8 ± 0.4, respectively (*p* < 0.01 for all comparisons). These findings confirm that ultrasonic-assisted nanoparticle processing not only enhances chemical composition but also significantly improves the sensory appeal of Saudi coffee in both cold and hot preparations.

## 4. Discussion

The analysis of Saudi coffee nanoparticles, prepared via an ultrasonic-assisted technique, reveals significant insights into their particle size, morphology, molecular structure, and sensory attributes. A particle size analyzer was employed to determine the size of Saudi coffee nanoparticles following the ultrasonication process. It emphasizes the importance of a uniform particle size distribution, a crucial factor for the functionality of nanoparticles in various fields such as drug delivery and food technology. The homogeneity of the particle distribution can be credited to multiple interconnected factors, such as improved dispersion from the ultrasonic processing [21]. Ultrasonication helps chemicals within the coffee matrix interact more easily, leading to decreased agglomeration and improved mixing efficiency. These procedures break down larger aggregates into smaller, uniform nanoparticles, enhancing the stability and functionality of the final product [12]. Reducing the particle sizes usually leads to a larger surface area for contact with solvents, which is beneficial for extraction procedures and the bioavailability of active compounds. The smaller size creates more surface sites for adsorption, which could improve the interaction with biological systems [22]. It was crucial to optimize ultrasonic parameters such as amplitude, power, and processing time for the effective manufacturing of Saudi coffee nanoparticles [23]. The results of this study highlight the importance of having precise control over these parameters to achieve the desired nanoparticle characteristics while upholding the structural integrity of bioactive compounds.

The surface morphology of the Saudi coffee nanoparticles was examined using SEM, offering detailed images at the nanometer scale. The irregular shapes suggest agglomeration caused by the intense energy of ultrasonic processing. SEM’s capability to offer in-depth observations enables a comprehensive evaluation of how processing methods impact particle shape [24]. Agglomeration of nanoparticles can impede their efficiency, especially in products that demand consistency like drug delivery systems. Although some agglomeration is present, the size and shape distributions remain suitable for effective nanoparticle systems. Nanoparticles with irregular shapes could increase the surface area and promote interactions with other materials, which may enhance their functional properties [25].

The examination of the molecular composition of the Saudi coffee nanoparticles via the FT-IR method revealed various functional groups such as hydroxyl (OH), alkane (CH), carbonyl (C=O), and aromatic compounds (C=C). These findings align with the results of past studies highlighting the presence of bioactive compounds in coffee that could offer advantages for health [26]. The O–H stretching vibrations indicate possible antioxidant properties, which are important in functional food uses. The nanoparticles’ ability to preserve these functional groups while undergoing the ultrasonic preparation process boosts their potential for pharmaceutical applications, where stability and bioactivity are crucial [24].

The markedly improved solubility of Saudi coffee nanoparticles reflects the critical role of nanoscale engineering in enhancing extractability. This aligns with previous reports highlighting how ultrasonic cavitation promotes structural breakdown and facilitates mass transfer during extraction. Increased surface area at the nanoscale improves solvent interaction, while pulsed ultrasonication prevents thermal degradation, thus preserving compound integrity [26]. Such improvements not only support higher concentrations of bioactive compounds like caffeine and phenolic compounds but also imply greater efficiency for applications in instant coffee and nutraceutical formulations. The rise in solubility enhances the extraction efficiency of active compounds and could also boost their bioavailability, a critical element in the creation of functional foods and nutraceuticals [27]. The pH reduction observed in Saudi coffee nanoparticles compared to the regular coffee extract may be attributed to the enhanced exposure and release of organic acids such as chlorogenic, citric, malic, and quinic acids, which are naturally present in coffee. The reduction in particle size increases the surface area, facilitating the liberation of these acids into the aqueous phase. Additionally, ultrasonic cavitation may promote mild hydrolysis or the degradation of larger phenolic compounds into smaller acidic fragments, further contributing to acidity. Although specific organic acids were not quantified in this study, future work will include the HPLC analysis of organic acid profiles to more precisely elucidate the mechanisms involved. Chemical reactions might take place while ultrasonication is happening, leading to the breakdown of specific acidic compounds, and reducing the pH even more [28]. The decrease in pH can improve the taste and shelf life of the coffee concentrate, resulting in increased consumer appeal.

The significantly elevated phenolic content observed in the nanoparticle formulation underscores the effectiveness of ultrasonic-assisted extraction in maximizing the yield of health-promoting compounds. The enrichment results from enhanced release and minimal degradation under controlled cavitation. From a functional food perspective, a higher phenolic content suggests enhanced antioxidant potential, which may contribute to the therapeutic and preventive health claims [29]. Furthermore, the notable rise in caffeine levels in nanoparticles compared to regular coffee suggests that the ultrasonic process improves caffeine extraction by increasing the surface area and enhancing interaction with the solvent. The high level of caffeine and phenolic content in Saudi coffee nanoparticles may provide significant health benefits, making them more appealing in the functional foods industry [30]. The sensory evaluation results indicate that Saudi coffee nanoparticles excel over normal coffee in multiple aspects, including aroma, taste, and overall impact. These results demonstrate the nanoparticles’ potential to improve the consumer sensory experience [31]. The improved sensory scores observed for Saudi coffee nanoparticles, particularly in terms of aroma, taste, and mouthfeel, can be supported by previous studies demonstrating that ultrasound-assisted extraction enhances the release of key volatile compounds, including pyrazines, furaneol, aldehydes, and phenols, which are known contributors to coffee flavor and aroma [32]. Ultrasonication facilitates cell wall disruption and increased mass transfer, which leads to the greater liberation of entrapped aroma-active molecules. This has been shown in coffee matrices and other plant-derived beverages, where ultrasonic processing resulted in a higher abundance of flavored volatile compounds and improved sensory characteristics. Therefore, it is likely that similar mechanisms contributed to the superior sensory performance of Saudi coffee nanoparticles in this study.

While ultrasonication and nanoparticle formation enhance the release of bioactive compounds such as caffeine and chlorogenic acid, over-extraction of these constituents may potentially lead to excessive bitterness or astringency. However, in this study, the ultrasonic parameters (amplitude, power, and time) were optimized to achieve particle size reduction and extraction enhancement without surpassing sensory acceptability thresholds. This is supported by the sensory panel ratings, which showed improved taste and aftertaste scores in the nanoparticle samples for both cold and hot coffee, with no indication of increased bitterness or harshness. These results suggest that the extraction remained within an optimal range that enhanced the yield of bioactive compounds while preserving or improving the overall sensory profile.

Enhanced sensory attributes, especially in warm drinks, could greatly impact consumer tastes and approval, suggesting a promising direction for commercial success. These findings suggest that coffee nanoparticles could improve the quality and sensory attributes of coffee, making it more competitive in the international market.

While direct GC–MS profiling was not performed, future studies should incorporate such analysis to better elucidate the molecular basis of flavor enhancement. Additionally, further research is needed to explore the potential applications of these nanoparticles in food and pharmaceutical formulations, and to understand their long-term effects on health and stability. Another limitation of the current study is the use of a sequential, stepwise approach for optimizing ultrasonic parameters. Although effective, this method lacks the statistical rigor of formal design-of-experiment strategies such as the Response Surface Methodology (RSM), which should be employed in future work to achieve more precise and reproducible optimization. The elevated levels of caffeine and phenolic compounds in the Saudi coffee nanoparticles suggest potential health-promoting properties, which may support their future application in the functional foods or nutraceutical industries. However, these findings are based on in vitro chemical characterization, and further in vivo studies and clinical evaluations are necessary to confirm their actual physiological effects and safety.

Nevertheless, further research is also necessary to explore the potential uses of these nanoparticles in food and pharmaceutical items, and to comprehend their long-term effects on health and stability.

## 5. Conclusions

This study confirms that ultrasonic-assisted nanoparticle preparation markedly improves the physicochemical and sensory properties of Saudi coffee. By reducing particle size and enhancing uniformity, the technique significantly increases solubility and facilitates more efficient extraction of key bioactive compounds, notably caffeine and phenolic compounds. These modifications not only improve the functional qualities of the coffee but also result in a richer sensory experience, including a better aroma, flavor, and mouthfeel. These enhancements position this method as a promising strategy for improving Saudi coffee’s extraction efficiency and sensory quality. Overall, ultrasonic nano-processing represents a valuable advancement in the development of high-quality, health enhancing coffee products with increased global appeal. However, the practical application of these nanoparticles in food and pharmaceutical systems remains contingent upon their long-term stability.

## Figures and Tables

**Table 1:** Comparison of physicochemical properties between Saudi coffee nanoparticles and regular Saudi coffee.

Parameter	Saudi Coffee Nanoparticles (Mean ± SD)	Regular Saudi Coffee (Mean ± SD)	*p*-Value *
pH	4.65 ± 0.07	5.76 ± 0.10	<0.001
Water Content (%)	4.37 ± 0.12	5.32 ± 0.15	<0.01
Solubility (%)	75.88 ± 5.25	40.70 ± 3.45	<0.001
Total Phenolic Compounds (μg GAE/g)	1825.3 ± 72.8	426.7 ± 48.4	<0.001
Caffeine (mg/g)	3.21 ± 0.09	0.54 ± 0.05	<0.001

* Statistical comparisons were performed using one-way ANOVA.

**Table 2:** Sensory evaluation of Saudi coffee nanoparticles versus regular Saudi coffee in both cold and hot preparations.

Sensory Attribute	Cold Coffee (Mean ± SD)			Hot Coffee (Mean ± SD)		
	Nanoparticles	Regular Coffee	*p*-Value *	Nanoparticles	Regular Coffee	*p*-Value *
**Aroma**	3.9 ± 0.3	2.5 ± 0.4	<0.01	4.2 ± 0.2	3.1 ± 0.3	<0.01
**Taste**	3.6 ± 0.4	2.3 ± 0.5	<0.01	4.0 ± 0.3	2.8 ± 0.4	<0.01
**Aftertaste**	3.4 ± 0.3	2.1 ± 0.4	<0.01	3.9 ± 0.3	2.7 ± 0.3	<0.01
**Acidity**	3.2 ± 0.4	2.0 ± 0.3	<0.01	3.7 ± 0.2	2.5 ± 0.3	<0.01
**Mouthfeel**	3.8 ± 0.3	2.3 ± 0.4	<0.01	4.1 ± 0.2	2.9 ± 0.3	<0.01
**Overall Impact**	3.6 ± 0.3	2.4 ± 0.4	<0.01	4.3 ± 0.2	2.8 ± 0.4	<0.01

* Statistical comparisons were performed using one-way ANOVA.

## Data Availability

Data sharing is applicable to this article.
